# Azide‐PEG‐Folate Functionalized Gadolinium Nanoparticles: A Preliminary Click‐Chemistry Platform for Magnetic Resonance Imaging Contrast

**DOI:** 10.1002/open.70276

**Published:** 2026-08-17

**Authors:** Retna Putri Fauzia, Azmi Aulia Rahmani, Zahra Afriani, Theresia Cicilia Magdalena, Cindy Florencia Sutanto, Galuh Yuliani, Angga Cipta Narsa, Safri Ishmayana, Ratna Dini Haryuni, Huanhuan Liu, Santhy Wyantuti

**Affiliations:** ^1^ Department of Chemistry Faculty of Mathematics and Natural Sciences Universitas Padjadjaran Sumedang Indonesia; ^2^ Chemistry Study Program Universitas Pendidikan Indonesia Bandung Indonesia; ^3^ Department of Pharmaceutics and Pharmaceutical Technology Faculty of Pharmacy Mulawarman University Samarinda Indonesia; ^4^ Research Center for Radioisotopes, Radiopharmaceuticals, and Biodosimetry Technology Research Organization for Nuclear Energy – National Research and Innovation Agency KST BJ Habibie Tangerang Selatan Indonesia; ^5^ Department of Radiology Henan Provincial People’s Hospital & the People’s Hospital of Zhengzhou University Zhengzhou P. R. China

**Keywords:** cancer, click reaction, diagnosis, folic acid, gadolinium nanoparticles

## Abstract

Gadolinium nanoparticles (GdNPs) have been studied extensively due to their lower toxicity and better magnetic resonance imaging (MRI) detection capabilities than Gd‐chelates. Their surfaces are conveniently modified with several molecules to enhance their accumulation in the cancer of interest, thereby enabling more accurate diagnosis and treatment. In this study, hydrothermal GdNPs were functionalized with folic acid (FA) (for active targeting), polyethylene glycol (to improve biocompatibility), and azide molecules (for subsequent click reaction) via a silane ligand exchange conjugation method. Results showed a hydrodynamic diameter of 7.90 nm and a zeta potential of −6.80 mV for Azide‐PEG‐Folate‐modified GdNPs. UV spectra confirmed the presence of FA in NPs at UV absorption of 284 and 353 nm. Typical FTIR spectra also appeared at wavenumbers of 2950 and 2150 cm^−^^1^, confirming the presence of PEG and azide molecules on modified NPs, respectively. Additionally, modified NPs maintained good stability for up to 3 weeks at 25 and 4 °C. A preliminary click in vitro investigation also showed the NPs’ good viability even after incubation at 132 µg/mL, followed by the click reaction with 300 µg/mL of Holmium(III)‐complexed‐DOTA‐BCN on HeLa cancer cells. This first click‐combination finding serves as a promising candidate for Gd/Ho‐MRI agents, thereby enabling a more accurate diagnosis due to their simultaneous T_1_/T_2_ contrast enhancement.

## Graphical abstract

The click‐modified targeted gadolinium nanoparticles are presented as a development of a multifunctional MRI contrast agent. The nanoplatform demonstrates improved stability and selective cellular targeting, and is expected to facilitate more precise tumor imaging while enabling further bioorthogonal conjugation for advanced cancer diagnosis and theranostic outcomes.

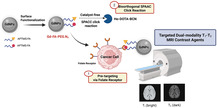

## 1 Introduction

Cancer remains a major global health challenge. The World Health Organization (WHO) estimates that 20 million new cases and 9.7 million deaths from cancer are reported annually worldwide. This number is expected to increase significantly, reaching almost 29 million cases by 2040 [1]. Effective cancer management requires not only improved therapies but also enhanced preventive strategies and early diagnostic approaches, as high mortality rates remain associated with limitations in early detection and treatment efficacy. The development of medical imaging modality technology is an important factor in early cancer detection and improved diagnostic accuracy. So far, there are widely used imaging modalities in modern clinical applications, including magnetic resonance imaging (MRI), computed tomography (CT), ultrasonography (US), single photon emission computed tomography (SPECT), and positron emission tomography (PET) [2]. Among these imaging modalities that have been used, MRI is considered to have its own advantages because it uses a magnetic field to activate protons [3], so that patients avoid exposure to ionizing radiation, and is able to provide high spatial and temporal resolution [4, 5, 6]. Compared to CT, PET, and US, MRI scans are better at soft tissue differentiation and allow for safer repeat imaging, making them highly suitable for the diagnosis and monitoring tumor progression [6, 7, 8, 9, 10].

The ability of MRI to distinguish soft tissue contrast is further enhanced when combined with contrast agents [11]. There are two types of MRI contrast agents, namely T_1_‐weighted contrast agents that produce images with bright contrast and T_2_‐weighted contrast agents that produce images with dark contrast [12]. Since the 1980s, more than 30% of MRI examinations have used contrast agents to improve tissue visualization [11]. The most commonly used MRI contrast agents are gadolinium chelates (GBCAs), which have been approved by the Food and Drug Administration (FDA) since 1988 [13]. Gadolinium is widely used because of its paramagnetic properties, arising from the 4f subshell’s seven unpaired electrons (*S* = 7/2) [14, 15, 16, 17, 18], which enhance contrast on T_1_‐weighted images.

However, although it has been observed that GBCAs are useful as contrast agents, it has also been noted that there are a number of limitations that are still of concern, such as the stability of the complex, a relatively short circulation time, and a potential increase of free Gd^3+^ ions that can lead to toxicity [19, 20, 21, 22]. Moreover, with GBCAs being administered to those with compromised renal function, there is a problem of nephrogenic systemic fibrosis, while there have been reports of retention of gadolinium in body tissues, particularly after repeated doses [23, 24, 25]. Therefore, there are significant opportunities to develop useful contrast agent systems with nanoparticles (NPs), as they are expected to enhance not only stability and biological safety but also efficiency, with greater controllability and more specific targeting potential [26, 27, 28, 29, 30].

The transformation of a gadolinium complex into NPs could allow for an increase in accumulation in tumor tissues via the enhanced permeability and retention effects, enabling accumulation in tumor tissues compared to normal tissues. In addition, due to their small size and high surface area, NPs can be functionalized with active targeting agents, such as folic acid (FA) [31, 32, 33, 34]. FA can have a high affinity for the folate receptor (FR) (Kd = 10^14^) [35, 36], which is overexpressed in a variety of different types of cancer cells, including cervical cell types, but not in normal cells [37]. In our previous study, (GdNPs) functionalized with FA showed higher internalization in cancer cells with high FR expression, such as HeLa and KB, compared to normal cells, suggesting potential as a targeted MRI contrast agent. Additionally, the NPs can have PEG moieties attached to prolong blood circulation without being recognized by the immune system [38, 39, 40, 41, 42].

However, both passive and active targeting have limitations, such as tumor heterogeneity, lack of differences among target receptors, and limited ligand‐binding affinity, which affect pretargeting efficiency [43, 44]. Therefore, a new approach for pretargeting using bioorthogonal click chemistry for efficient molecular conjugations without interfering with biological systems has emerged [45, 46]. The click reaction was first introduced by Sharpless et al. In 2001 as a selective reaction that is fast, efficient, and compatible with simple reaction conditions compared to traditional synthesis [47]. The main advantage of this reaction lies in its bioorthogonal nature, in which the precursor is inert toward other biological functional groups (e.g., amines, carboxylic acids, and thiols), thereby minimizing side reactions [48, 49]. The click reaction offers fast kinetics in aqueous media, high selectivity, and minimal byproducts [50, 51, 52]. Its bioorthogonal nature allows the reaction to proceed without disrupting the natural functional groups in biological systems, making it ideal for in vivo applications [53]. Although copper‐catalyzed alkyne‐azide cycloaddition (CuAAC) is a classic example, the use of copper as a catalyst is limited by its toxicity; therefore, highly strained cyclooctyne derivatives are widely used because they can react in the absence of a metal catalyst [54]. Strain‐promoted azide‐alkyne cycloaddition (SPAAC) has evolved into a Cu‐free reaction that uses strained alkynes to rapidly and efficiently link molecules. For example, the cycloaddition reaction between bicyclooctyne (BCN) and azide, developed by Bertozzi et al. In 2010 is inert toward other biomolecular groups and exhibited a high reaction rate (*k* = 2.0–2.9 M^−1^s^−1^), making it ideal for in vivo application [47, 55].

Furthermore, the introduction of azide (N_3_) groups on the NP surface provides bioorthogonal sites that enable a two‐step targeting strategy via click chemistry reactions, thereby facilitating selective and modular conjugation with ligands or additional imaging agents [56]. This approach enables a modular, bioorthogonal conjugation strategy, in which NPs can be specifically linked to cyclooctyne molecules carrying diagnostic or therapeutic payloads, potentially forming an integrated nanotheranostic platform [57].

In this research, we explored Ln(III)‐based cyclooctyne complexes, such as Ho(III), as a preliminary study of click reactions with modified‐GdNPs. Click reactions were tested using 1,4,7,10‐tetraazcyclododecane‐1,4,7,10‐tetraacetate (DOTA) derivatives modified with cyclooctyne (BCN) complexed with Ln(III), with Ho‐DOTA‐BCN as a model molecule. The combination of Gd and Ho as T_1_–T_2_ dual‐mode MRI contrast offers the opportunity to develop enhancement of positive‐bright and negative‐dark MRI visualization, thereby improving diagnostic accuracy [58]. The present study develops a promising strategy in which Gd‐based T_1_ NPs and Ho‐DOTA T_2_ contrast agents are conjugated through a click‐chemistry approach. The design allows NPs to be individually optimized prior to conjugation while maintaining their magnetic properties or imaging capabilities.

Recent advances in MRI diagnostic contrast enhancement through dual T_1_/T_2_ modality have been explored with the utilization of magnetic nanomaterials, such as Gd, Mn, Fe, or other lanthanide metals, to integrate both imaging modalities [58]. Nevertheless, the click chemistry T_1_–T_2_ integration system has not been extensively investigated; the design must still be further evaluated in order to optimize its further MRI‐relaxivity.

In the future, this click‐chemistry‐based pretargeting strategy has the potential to be developed into a targeted delivery system for radionuclides and chemotherapeutic agents, not only to improve the precision of cancer diagnosis, but also for successful treatment purposes.

**SCHEME 1:**
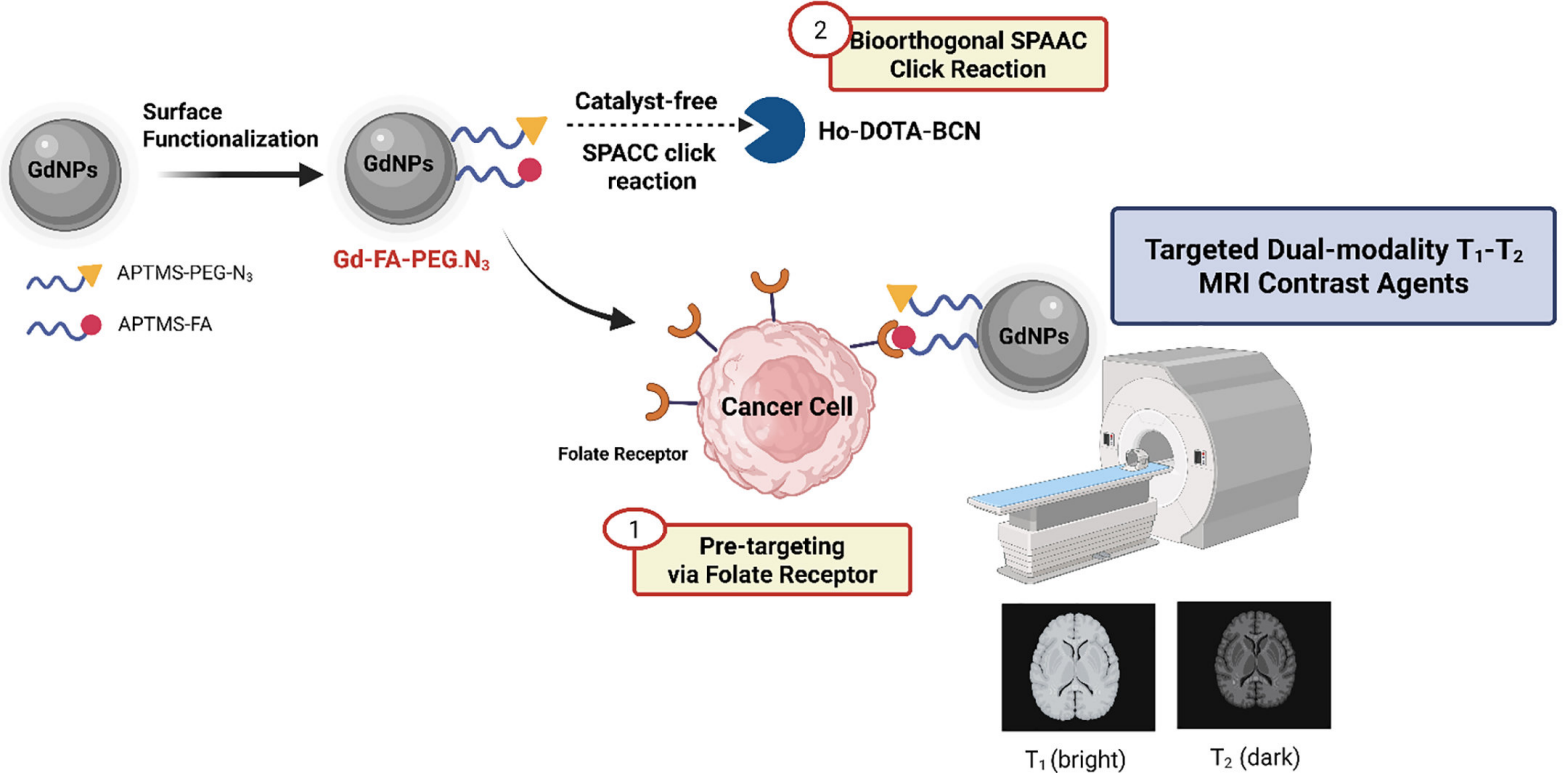
Schematic illustration of click chemistry functionalization and conjugation strategies in the design of folic acid‐targeted dual‐modality (T_1_–T_2_) MRI contrast agents.

In this study, GdNPs were hydrothermally synthesized and functionalized with active targeting ligands, such as FA, PEG to improve biocompatibility, and azide groups to provide bioorthogonal reactive sites for click chemistry (Scheme 1). In addition, a preliminary study of the click reaction was conducted between NPs containing azide groups and cyclooctyne molecules modified with DOTA and complexed with Ho(III) ions. The combination of gadolinium, a T_1_ contrast agent, and holmium, a T_2_ contrast agent, is expected to produce a multimodal T_1_–T_2_ contrast agent that could improve the sensitivity and accuracy of MRI‐based diagnostic imaging.

## 2 Results and Discussion

### 2.1 Functionalization of GdNPs With APTMS‐FA and APTMS‐PEG‐N_3_

Spherical GdNPs with a diameter of 6.60 ± 0.30 were previously synthesized via the hydrothermal method based on Wyantuti et al. [30]. The hydrothermal approach employed high temperatures and pressures, using a direct crystallization mechanism from solution and comprising two main stages: nucleation and crystal growth, in order to achieve NP formation. Polyethylene glycol‐6000 (PEG_6000_) was added as a stabilizer to prevent aggregation by interacting with the gadolinium NP surface [12, 30, 34].

**FIGURE 1:**
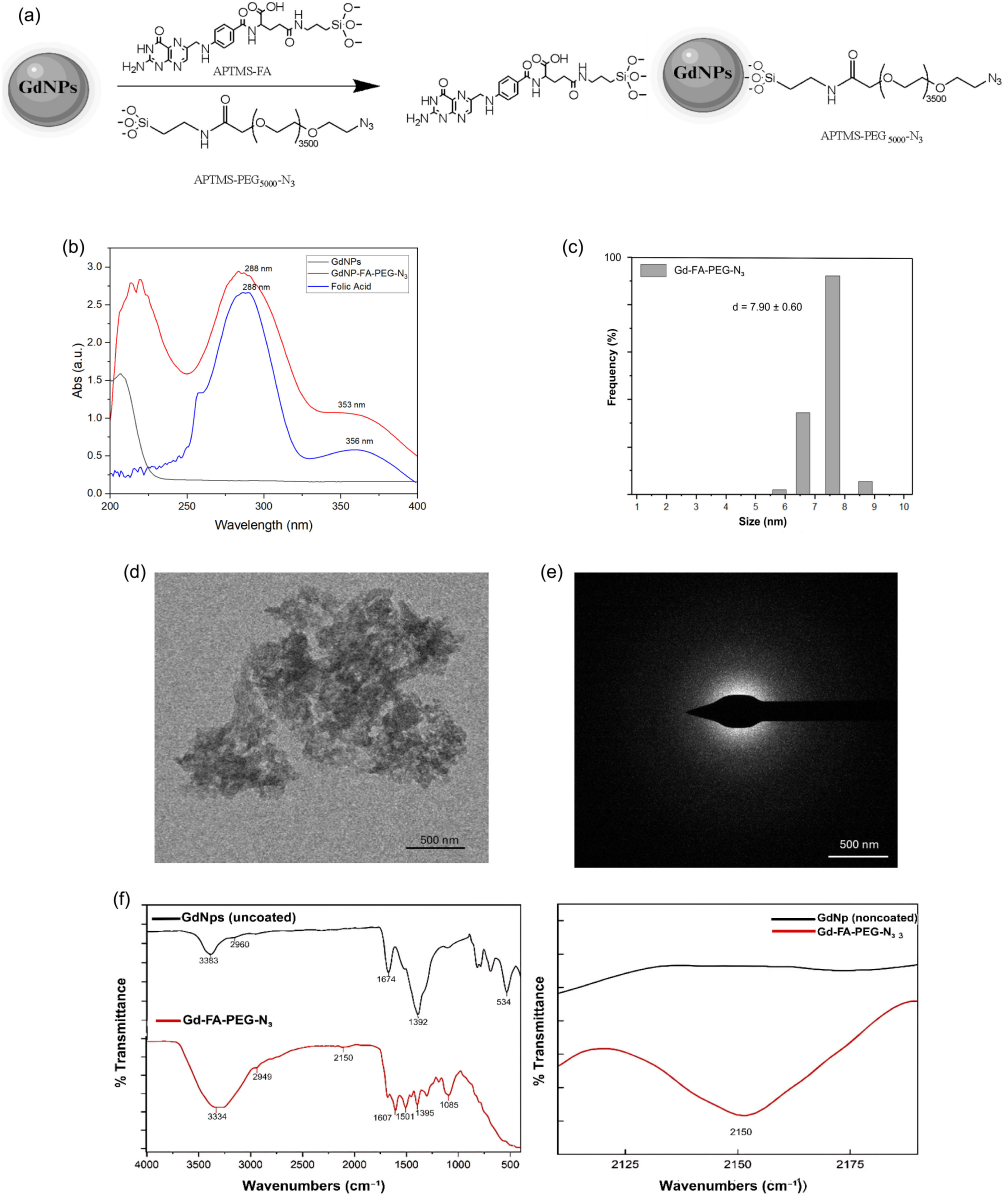
Schematic illustration of (a) attachment of APTMS‐FA and APTMS‐PEG‐N_3_ on GdNPs (b) UV–vis and (c) DLS (d and e) TEM images, and (f) FTIR results of GdNPs (uncoated) and Gd‐FA‐PEG‐N_3_ (red).

Furthermore, GdNPs were then functionalized with FA and polyethylene glycol‐azide (PEG‐N_3_) molecules. FA served as an active targeting agent, enabling the specific delivery of NPs to cancer cells that overexpress FRs, such as those found in cervical cancer [34, 59, 60]. PEG was used to enhance biocompatibility and prevent interparticle agglomeration [61, 62, 63]. At the same time, the azide group at the terminal end of PEG enabled further applications through click reactions, which were helpful for multimodal imaging and theranostic purposes (Figure 1a).

In the functionalization process, FA and PEG‐N_3_ were first conjugated with the organosilane molecule (3‐aminopropyl)trimethoxysilane (APTMS), which contained a reactive amine group that allowed it to serve as an intermediate for the incorporation of various functional molecules, including FA and PEG‐N_3_ [23]. The APTMS‐FA and APTMS‐PEG‐N_3_ conjugates were then grafted onto the NP surface through a process known as silanization, which involved the modification of the NP surface with silane molecules via the formation of covalent bonds between the alkoxysilane group (─Si─OR) of APTMS and hydroxyl groups on the NP surface (Figure 1a) [64]. APTMS‐FA and APTMS‐PEG‐N_3_ were synthesized via coupling reactions using 1‐ethyl‐3‐(3‐dimethylaminopropyl)‐carbodiimide hydrochloride (EDC) and N‐hydroxysuccinimide (NHS). In the synthesis of APTMS‐FA, FA was first activated to NHS‐FA using EDC and then reacted with APTMS to form APTMS‐FA (Figure S1). Meanwhile, for the synthesis of APTMS‐PEG‐N_3_, PEG‐N_3_ in the form of an active ester (NHS‐PEG‐N_3_) was used, allowing direct reaction with APTMS to form APTMS‐PEG‐N_3_.

The success of functionalization of GdNPs was confirmed by ultraviolet (UV) and Fourier transform infrared (FTIR) spectroscopy, which verified the presence of PEG and FA molecules on the surface of GdNPs. The UV spectra characterization results in Figure 1b showed the presence of two typical absorption peaks of FA molecules at wavelengths around 284 and 353 nm, which correspond to the π → π* and n → π* transitions of the aromatic and carbonyl groups in FA. These peaks were detected in the Gd‐FA‐PEG‐N_3_ sample but were not observed in GdNPs (uncoated), indicating that FA conjugation had been successfully achieved. Meanwhile, the FTIR spectrum of Gd‐FA‐PEG‐N_3_, as shown in Figure 1f, exhibited characteristic absorption bands of PEG groups at 2949 and 3334 cm^−1^. In addition, the presence of the azide group (N_3_) was confirmed by the appearance of a typical N_3_ absorption band at 2150 cm^−1^ in Gd‐FA‐PEG‐N_3_ spectra, which did not appear in GdNPs spectra, indicating that the N_3_ group had also been successfully conjugated.

Furthermore, the morphology of the Gd‐FA‐PEG‐N_3_ was analyzed by thermal emission microscope (TEM), as shown in Figure 1d,f. TEM results indicate that, after the functionalization, NPs showed a greater tendency to aggregate, forming denser agglomerates than those observed for the GdNPs. This suggests that surface modification via the attachment of functional molecules increases interparticle interactions, probably as a consequence of the interaction of PEG chains or the presence of FA on the surface of GdNPs, although the structure of their core is preserved. These findings are consistent with results from dynamic light scattering (DLS) measurements (Figure 1d and Table 1), which showed an increase in the hydrodynamic size of the GdNPs from 6.60 ± 0.30 nm prior to conjugation to 7.90 ± 0.60 nm after functionalization. This increment indicated the attachment of functional molecules to the NP surface, leading to an increase in hydrodynamic diameter due to the formation of a surrounding molecular layer. Overall, the size of the NPs, which remains sub‐20 nm, is regarded as an important factor as it enables effective tissue penetration and favorable biodistribution [65]. In addition, the smaller particle size helps improve the access of water molecules to the gadolinium ions, which can consequently improve their longitudinal relaxivity (r_1_) and thus efficiency as a T_1_‐contrast agent [12, 61].

**TABLE 1:** Characterization of Gd‐FA‐PEG‐N_3_ nanoparticles using DLS and zeta potential.

Nanoparticles	Size, nm	Zeta potential, mV
GdNPs	6.60 ± 0.30	+36.7 ± 0.80
Gd‐FA‐PEG‐N_3_	7.90 ± 0.60	−6.80 ± 0.1

According to the Solomon–Bloembergen–Morgan Theory [66], longitudinal relaxivity (r_1_) is influenced by several parameters, such as the rotational motion of the NPs, the exchange rate of water molecules correlated to the metal ions, and their association with ion centers. Thereby, the moderate increase in NPs’ surface due to their modification can improve the rotational motion and subsequently enhance the T_1_‐relaxivity. However, an extreme aggregation of NPs may decrease the water accessibility to the surface of the metal ion, hence delaying the water exchange and reducing the T_1_‐contrast imaging result. Nevertheless, quantitative relaxivity must be further investigated to prove the NPs’ surface relationship with their MRI properties [67].

The zeta potential measurement of Gd‐FA‐PEG‐N_3_ showed an average value of −6.80 ± 0.1 mV within the range of −20 to 20 mV (Figure 5), indicating that the NPs had good colloidal stability due to electrostatic repulsion between particles. This negative surface charge was likely due to ─OH groups bound to the NP surface during PEG functionalization. In general, the surfaces of cells and blood components are negatively charged, so negatively charged NPs will experience minimal electrostatic interactions with these components, thereby prolonging circulation time and reducing clearance by the mononuclear phagocyte system [68].

### 2.2 Stability Study of Gd‐FA‐PEG‐N_3_

Among the important issues affecting NP behavior and interaction with cell organelle structures in the medium, stability plays an essential role. Colloidal instability may result in protein aggregation and adsorption. Finally, this may result in an increased particle size and decreased activity of NPs in drug‐associated therapies. The stability of NPs in a biochemical medium in the body is affected by medium properties, such as pH and ionic environment, and NP properties, which involve size, charge, and nature of surface coatings. FA‐functionalized GdNPs (GdNP‐FA‐PEG‐N_3_) were synthesized and stored in a medium over an extended period of 1 month to evaluate stability by observing variations in particle size, surface charge (Zeta potential), and the amount of FA adsorbed onto the NPs. PBS medium was used with an adjustment of the pH to 7.4 to mimic the natural environment of physiological systems. This was done in order to ensure that variations in the physicochemical properties of NPs precisely reflect NP stability in the medium.

**FIGURE 2:**
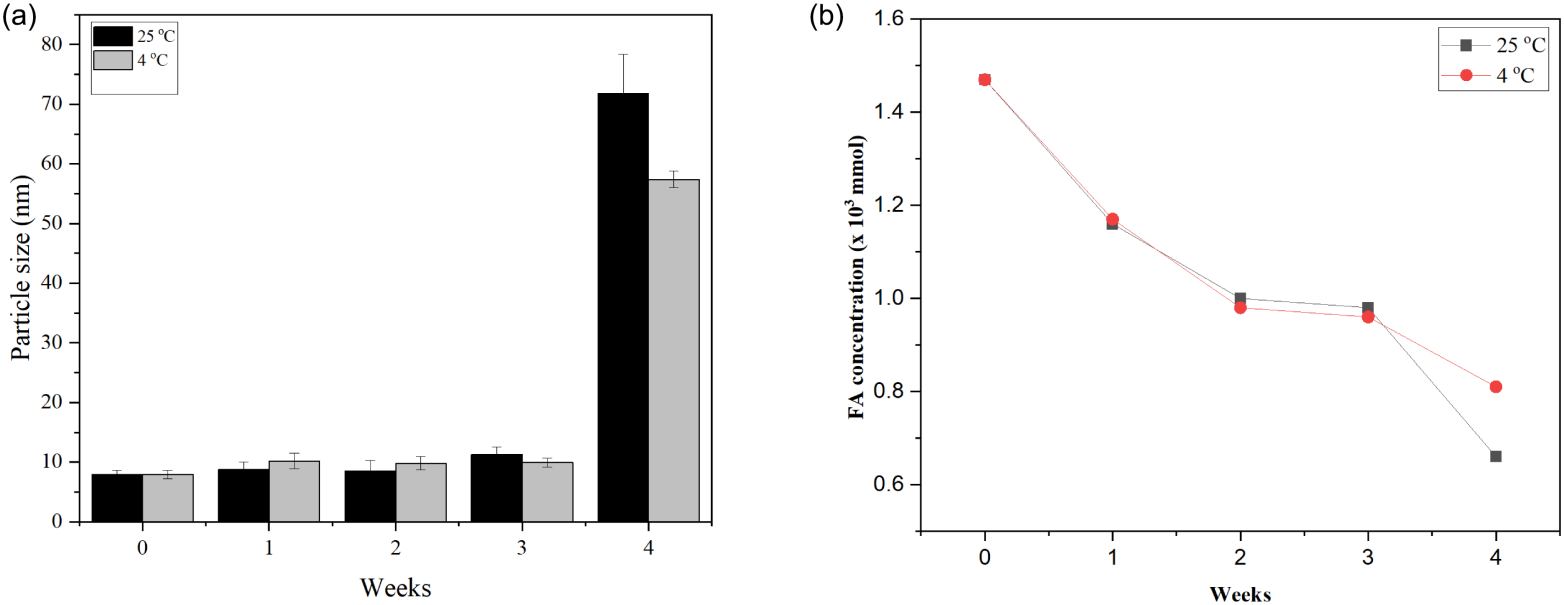
Stability of nanoparticles during 4 weeks of storage: (a) change in hydrodynamic size of nanoparticles; and (b) folic acid concentration as a function of time.

Figure 2a presents the results of the analysis, which shows that storage temperature is an important variable in the stability of NP size, but does not prevent colloidal instability altogether. Thus, in the fourth week at 25 °C, a significant increase in NP size was evidence of accelerated particle aggregation. This may be attributed to increased kinetic energy of the particles at this temperature, which increases the frequency of inter‐NP collisions, which consequently weakens both electrostatic and steric forces of stabilization, hence leading to a hydrodynamic size increase. Conversely, a lower temperature of 4 °C has kept the size of NPs relatively stable in the first 3 weeks, showing that such a temperature can delay aggregate formation by a decrease in particle mobility and rate of inter–NP interactions (Figure S1). However, in the fourth week, the sudden increase in size shows that mechanisms of degradation or instability proceed slowly but gradually after all and at a slower pace compared to room temperature. It may be that at this temperature, reorganization of the PEG layer, weakening of ligand–surface interactions, or ionic effects of the storage medium occur. The optimization of PEG density can also enhance NPs’ stability and prevent their aggregation [69].

Results from the test confirmed a significant difference in size between week 0 and week 4 at both temperatures (*p* < 0.05) (Table S3 and S4). From the results obtained in this study, it can be concluded that there is a relative advantage of NP size stability at low temperatures. However, there was a significant effect on size after storing for a month, as shown by the results from weeks 0 and 4 at both temperatures.

In addition, from a translational perspective, long‐term stability is crucial for further MRI applications of nanomaterials. The tendency of NP aggregation may alter the hydrodynamic size, blood circulation time, cellular uptake, and MRI relaxivity specifically [70]. Accordingly, their physicochemical stability still must be further investigated to enable safe clinical translation of NPs.

Apart from particle size stability, the stability of FA conjugation at the NP surface is another important factor affecting the success of an active targeting system. The possible changes in FA concentration could reflect the release of the ligand, chemical degradation, or structural change in the surface layer of NPs during storage and thus may affect the ability to target FRs.

The FA content of the NPs was quantified using a UV spectrophotometer, and the outcomes are reflected in Figure 2b. The results showed that at both 25 and 4 °C, the amount of FA conjugated to the NP surface decreased with storage time (Figure S2). The FA concentration decreased from 1.16 ± 0.18 × 10^3^ mmol at the beginning of the week to 0.66 ± 0.16 × 10^3^ mmol over the fourth week at 25 °C. On the contrary, at 4 °C, this decline went from 1.17 ± 0.19 × 10^3^ mmol to 0.81 ± 0.77 × 10^3^ mmol within the same period. These studies suggest that low‐temperature storage conditions more easily maintain the stability of FA conjugates and might be able to maintain the targeting ability of NPs before use in biological systems.

### 2.3 UV‐Click Reaction Monitoring

**FIGURE 3:**
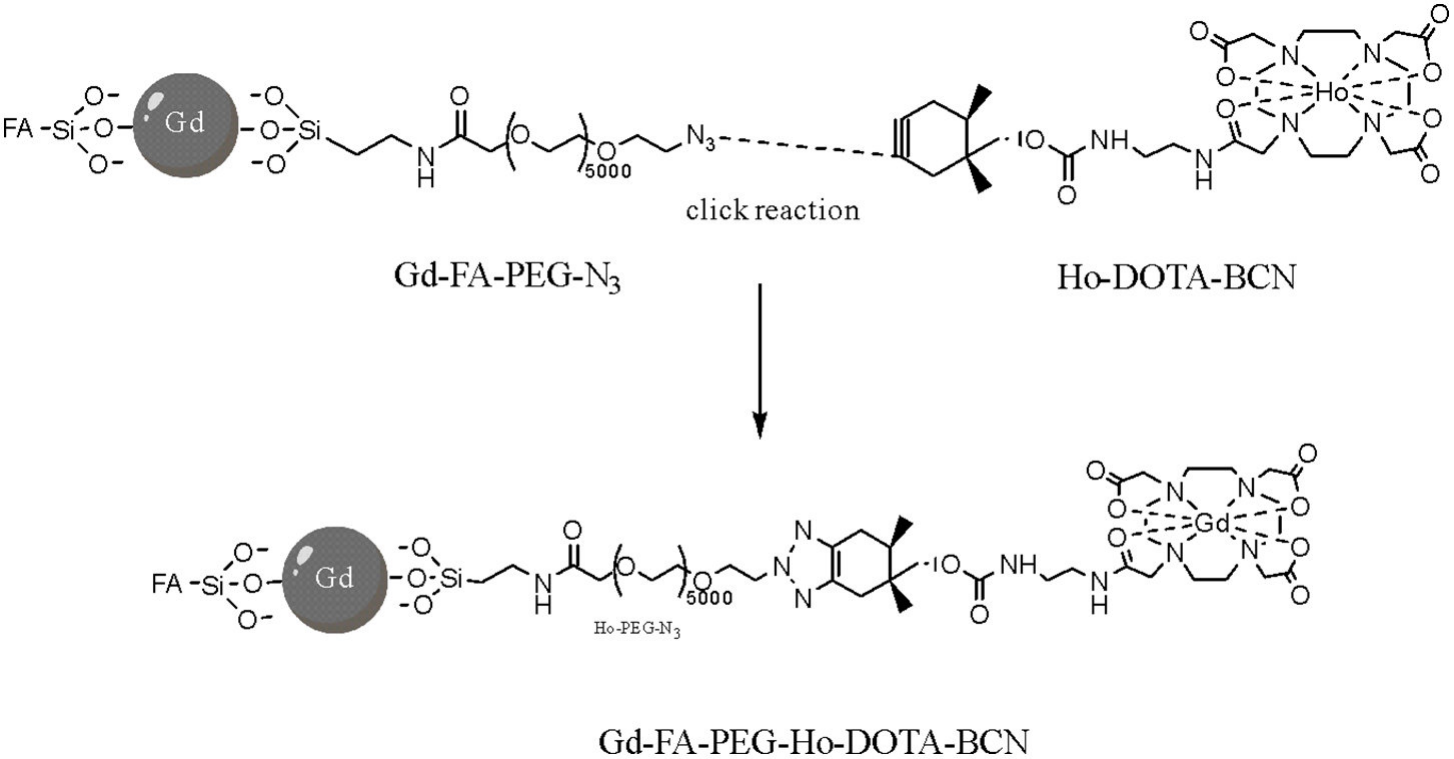
Schematic of the proposed click reaction between Gd‐FA‐PEG‐N_3_ nanoparticles and Ho‐DOTA‐BCN forming a triazole ring.

1,4,7,10‐tetraazacyclododecane‐1,4,7,10‐tetraacetate (DOTA) was modified with cyclooctyne (BCN) and complexed with holmium (Ho), forming the Ho‐DOTA‐BCN complex (Figure 3). DOTA was selected as the complexation ligand for Ho(III) due to its high thermodynamic and kinetic stability compared to other linear ligands. This complexation was also essential to prevent potential charge interference from free carboxylic groups [25].

**FIGURE 4:**
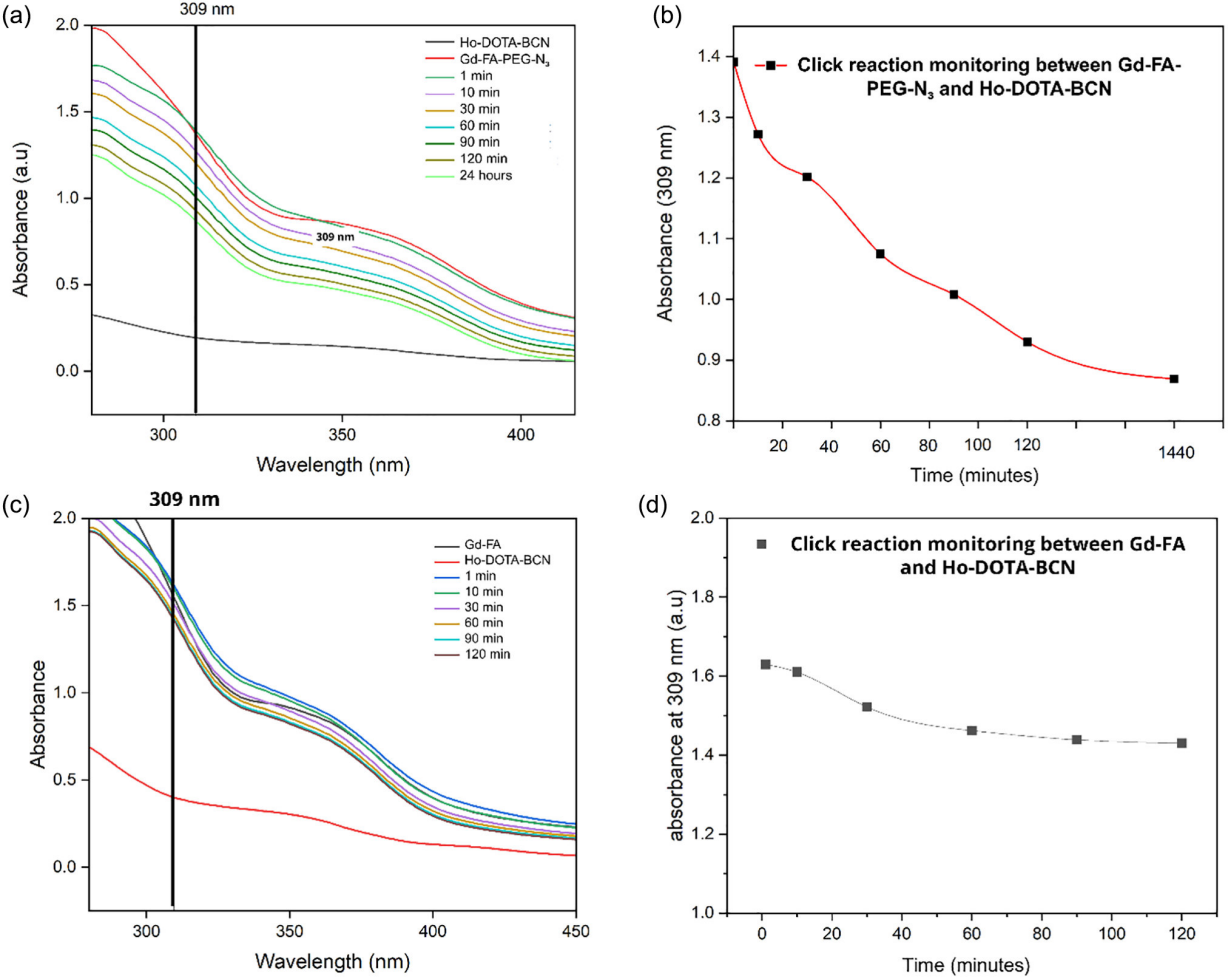
UV spectra of click reaction between (a,b) Gd‐FA‐PEG‐N_3_ nanoparticles with Ho‐DOTA‐BCN (c,d) Gd‐FA nanoparticles with Ho‐DOTA‐BCN.

The click reaction involved the formation of a covalent bond between the azide group (N_3_) and the cyclic alkyne group (BCN), resulting in the formation of a triazole ring. This reaction was confirmed using UV spectrophotometry, which showed a typical decrease in absorbance at a wavelength of 309 nm following the formation of triazoles [71]. The UV measurement results shown in Figure 4a,b demonstrated a significant decrease in absorbance at a wavelength of 309 nm after Gd‐FA‐PEG‐N_3_ was reacted with Ho‐DOTA‐BCN. These indicated the success of the click reaction and the formation of the triazole ring, which appeared after 1 min of reaction. As a control, gadolinium‐functionalized folic acid (Gd‐FA) NPs lacking azide groups were also reacted with Ho‐DOTA‐BCN. The UV spectra in Figure 4c,d did not show any significant decrease in absorbance at a wavelength of 309 nm. These results indicated that the click reaction does not occur if the NPs are not functionalized with azide groups.

Overall, these first results suggested that the presence of azide groups on the NP surface was a critical requirement for the click reaction with Ho‐DOTA‐BCN to occur specifically and efficiently. For further application, this click‐combination also enabled the use of the NPs as dual‐contrast (T_1_–T_2_) agents by combining the T_1_ contrast properties of gadolinium with the T_2_ contrast capabilities of holmium, thereby enhancing sensitivity and accuracy in medical diagnostic applications.

Unlike conventional dual‐mode T_1_–T_2_ MRI contrast, the proposed strategy separates the synthesis of each MRI platform component before bioorthogonal conjugation. The design allows Gd‐based NPs and the Ho‐DOTA component to be optimized first prior to combination; hence, their magnetic properties are expected to be maintained, and it offers the flexibility for future incorporation with another imaging probe or targeting ligand.

### 2.4 Cell Viability Click Reaction

Cell viability observation was carried out using the in vitro resazurin reduction assay. This assessment depends on the metabolic activity of the cells, i.e., the capability of the cells to reduce the nonfluorescent resazurin to the fluorescent resorufin [72, 73]. The resazurin reduction assay was used to assess the biocompatibility of the GdNP‐FA‐PEG‐N_3_ NPs with and without the bioorthogonal click reaction with the Ho‐DOTA‐BCN complex in HeLa cells. HeLa cell model was chosen due to their enormous FA receptor overexpression, enabling the specific interaction of FA‐coated NPs [74, 75, 76].

**FIGURE 5:**
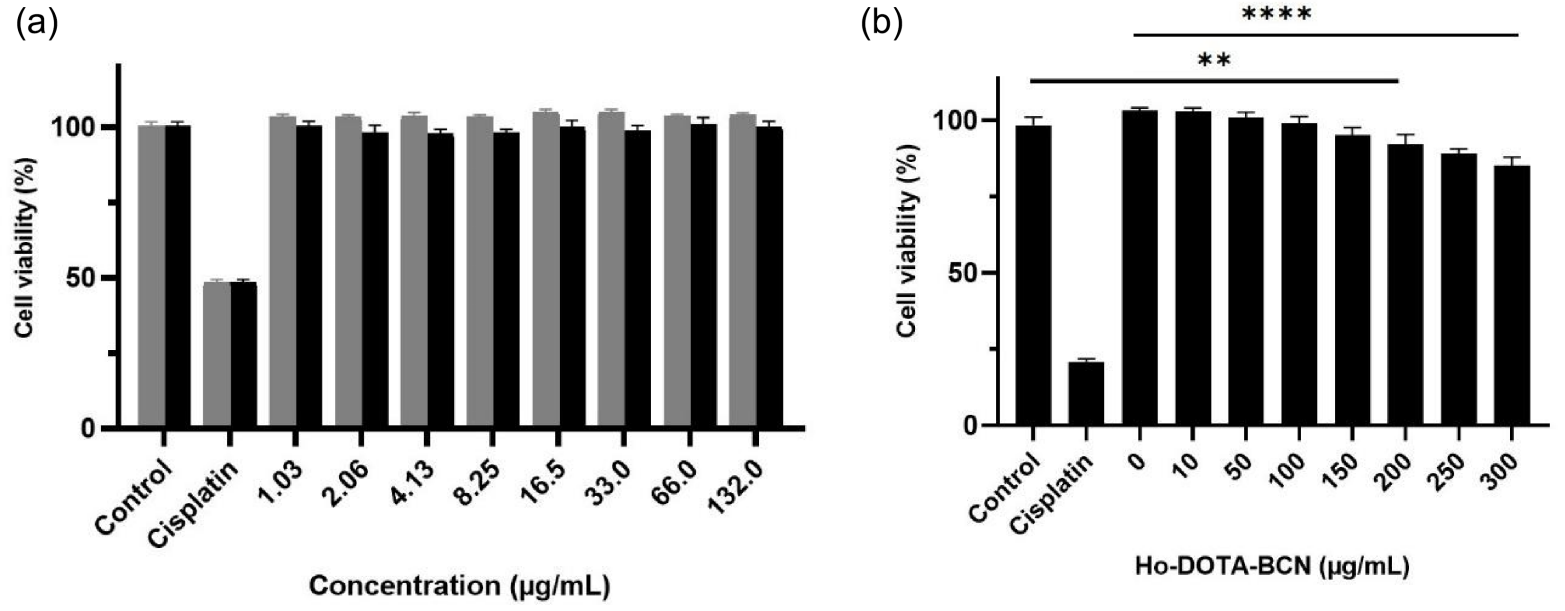
Preliminary in vitro cytotoxicity of (a) GdNPs (gray) and GdNPs‐FA‐PEG‐N_3_ (black) before and (b) after click reactions with the Ho‐DOTA‐BCN in the HeLa cell line. Viability was assessed using the resazurin reduction assay following 24 h of treatment (*n* = 4) (**p < 0.001 and ****p < 0.0001).

The resazurin reduction assay data in Figure 5a reveal that the viability of HeLa cells treated with the GdNP‐FA‐PEG‐N_3_ NPs for 24 h was maintained at a relatively high concentration of 132 µg/mL compared to the control, which was >90%. This revealed that the NPs were biocompatible and did not cause any disruption in the metabolic activity of the cells. In addition, the PEG molecules on the NP surfaces may improve the colloidal stability of the NPs and reduce nonspecific interactions with the cell membrane, thus reducing the potential for any cytotoxic effects.

After the initial incubation with GdNP‐FA‐PEG‐N_3_ NPs, Ho‐DOTA‐BCN complex was added with gradually increasing concentrations of Ho‐DOTA‐BCN (0, 10, 50, 100, 150, 200, 250, and 300 μg/mL) and incubated for another 24 h to assess the effect of the in situ click reaction on cell viability. The results revealed that the cell viability after the click reaction was still >80%. However, a significant decrease in viability after the click reaction was observed at a concentration of 200 μg/mL (*p* < 0.0001), indicating a concentration effect on the cellular response after the click reaction. This suggests that the click reaction between the azide group of the NPs and the DOTA‐BCN occurred without affecting cell metabolism. This may be because the bioorthogonal strain‐promoted azide‐alkyne cycloaddition reaction is metal‐free, which is safer and more desirable for biological applications. Based on these results, the developed GdNP‐FA‐PEG‐N_3_ NPs are expected to be a safe MRI contrast agent with superior functionalization capabilities mediated by bioorthogonal click reactions.

## 3 Conclusion

In conclusion, GdNPs were successfully functionalized with FA for active targeting, PEG to improve biocompatibility and colloidal stability, and azide groups that enable bioorthogonal click reactions with the Ho‐DOTA‐BCN complex. The presence of FA was confirmed through characteristic absorption at 284 and 353 nm (UV–vis), while the presence of PEG‐N_3_ was identified through FTIR spectra showing N≡N stretching bands at 2150 cm^−1^ and C─H stretching bands at 2950 and 2830 cm^−1^. DLS analysis showed a hydrodynamic diameter of 7.90 ± 0.60 nm with a zeta potential of −6.8 ± 0.1 mV, indicating successful surface modification while maintaining adequate nanoscale size and stability. Furthermore, Gd‐PEG‐FA‐N_3_ exhibited good stability for up to 3 weeks at 25 and 4 °C, indicating adequate colloidal stability for biomedical applications. Nevertheless, to enable safe clinical translation, its long‐term stability must be further investigated. To prove the concept of the click reaction, UV–vis monitoring demonstrated rapid triazole ring formation, characterized by a characteristic absorption decrease at 309 nm in less than 1 min. Initial in vitro click investigations also demonstrated good biocompatibility, where the NPs maintained high cell viability even after incubation at a concentration of 132 µg/mL Gd‐PEG‐FA‐N_3_, followed by a click reaction with 300 µg/mL Holmium(III)‐DOTA‐BCN complex in HeLa cancer cells. These findings demonstrate the success of the first click combination between Gd‐PEG‐FA‐N_3_ and Ho‐DOTA‐BCN as a promising candidate for Gd/Ho‐based dual‐modality MRI agents, with the potential to improve diagnostic accuracy through simultaneous T_1_/T_2_ contrast enhancement.

## Funding

This study was supported by Universitas Padjadjaran through *Riset Kolaborasi Indonesia* Scheme (Contract No. 4789/UN6.3.1/PT/.00/2025).

## Conflicts of Interest

The authors declare no conflicts of interest.

## Supporting information

The authors have cited additional references within the Supporting Information.

## Data Availability

The data that support the findings of this study are available on request from the corresponding author. The data are not publicly available due to privacy or ethical restrictions.
