# Supplementary material for: Azide‐PEG‐Folate Functionalized Gadolinium Nanoparticles: A Preliminary Click‐Chemistry Platform for Magnetic Resonance Imaging Contrast
Source: ChemistryOpen. 2026 Aug 17;15(9):e70276. doi: 10.1002/open.70276 (PMC13482026; doi:10.1002/open.70276)
Supplement: Supplementary file 1 [file OPEN-15-e70276-s001.pdf]

Supporting Information for

AZIDE-PEG-FOLATE FUNCTIONALIZED GADOLINIUM NANOPARTICLES: A PRELIMINARY CLICK-CHEMISTRY PLATFORM FOR MAGNETIC RESONANCE IMAGING CONTRAST

Retna Putri Fauzia *[a], Azmi Aulia Rahmani [a], Zahra Afriani [a], Theresia Cicilia Magdalena [a], Cindy Florencia Sutanto [a], Galuh Yuliani [b], Angga Cipta Narsa [c], Safri Ishmayana [a], Ratna Dini Haryuni [d], Huanhuan Liu [e], Santhy Wyantuti [a]

[a] R. P. Fauzia*, A. A. Rahmani, Z. Afriani, T. C. Magdalena, C. F. Sutanto, S. Ishmayana, S. Wyantuti
Department of Chemistry, Faculty of Mathematics and Natural Science
Universitas Padjadjaran
Jl. Raya Bandung-Sumedang Km. 21 Jatinangor, Sumedang 45363, Indonesia
E-mail: retna.fauzia@unpad.ac.id

[b] G. Yuliani
Department of Chemistry Education,
Universitas Pendidikan Indonesia,
Bandung 40154, Indonesia

[c] A. C. Narsa
Department of Pharmaceutics and Pharmaceutical Technology, Faculty of Pharmacy
Mulawarman University
Samarinda, 71157, Indonesia

[d] R. D. Haryuni
Research Center for Radioisotopes, Radiopharmaceuticals, and Biodosimetry Technology,
Research Organization for Nuclear Energy - National Research and Innovation Agency, KST BJ Habibie
Tangerang Selatan 15314, Indonesia

[e] H. Liu
Department of Radiology, Henan Provincial People's Hospital & the People's Hospital of Zhengzhou University
Zhengzhou, 450003 P. R. China

Table of Contents

Materials : ... 2
Instruments : ... 2
Functionalization of Gadolinium Nanoparticles : ... 2
Characterization of Gadolinium Nanoparticle Before and After Functionalization : ... 3
Stability test : ... 3
Synthesis of Ho-DOTA-BCN Complex ... 3
Monitoring of Click Reaction ... 3
Cell Culture ... 4
Cell Viability Click Reaction ... 4
Statistical Analysis ... 4

Materials :

1,4,7,10-tetraazacyclodododecane-1,4,7,10-tetrasethate-cyclooctane(DOTA-BCN), 1-ethyl-3-(3-dimethylaminopropyl)carbodiimide hydrochloride (EDC), (3-aminopropyl)trimethoxysilane (APTMS), dimethyl sulfoxide (DMSO, Merck), demineralized water, ethanol (Sigma Aldrich), folic acid (Sigma Aldrich), hydrochloric acid, glacial acetic acid (Merck, 100%), gadolinium chloride hexahydrate ($GdCl_3$. 6H2O, >99.9%), gadolinium oxide (Gd_2O_3, >99.99%), holmium chloride hexahydrate (HoCl3. $6H_2O$, >99.9%), milli-Q water, mtt test reagent, nitric acid (Sigma Aldrich, 65%), N-Hydroxysuccinimide (NHS, Sigma Aldrich), NHS-PEG_{5000}-azide ester, sodium acetate (CH_3COONa, Merck), sodium dodecyl sulphate (SDS), sodium hydroxide (NaOH, >99.9%), polyehtlene glycol-6000 (PEG_{6000}), Phosphate Buffered Saline (PBS), Roswell Park Memorial Institute (RPMI) medium, and silica gel.

Instruments :

Zeta potential measurements were carried out on Malvern Zetasizer NanoZs, Dynamic Light Scattering, Fourier Transform Infrared measurements were carried out on Thermo Scientific FTIR Lite ATR ZNSE, and UV-Vis Spectra were measured on LAMBDA 365+ Uv/Vis.

Functionalization of Gadolinium Nanoparticles :

Gadolinium nanoparticles (GdNPs) were synthesized hydrothermally by a similar method to that reported by Wyantuti et al. 2023 [1]. For functionalization of GdNPs, two different conjugates were used, namely, folic acid-conjugated silane (APTMS-FA) and polyethylene glycol-azide-conjugated silane (APTMS-PEG-N_3).

To prepare APTMS-FA, 0.1 mmol FA was dissolved in 1 mL of dry DMSO, 0.2 mmol of EDC was added, and the mixture was stirred for 3 hours, followed by 0.1 mmol of NHS. This mixture was then allowed to react for 24 hours at room temperature in the dark to solve activated folic acid (NHS-FA), which was then reacted with 0.1 mmol of APTMS for 24 hours to produce APTMS-FA. The obtained compound was then characterized using a UV-Vis spectrophotometer.

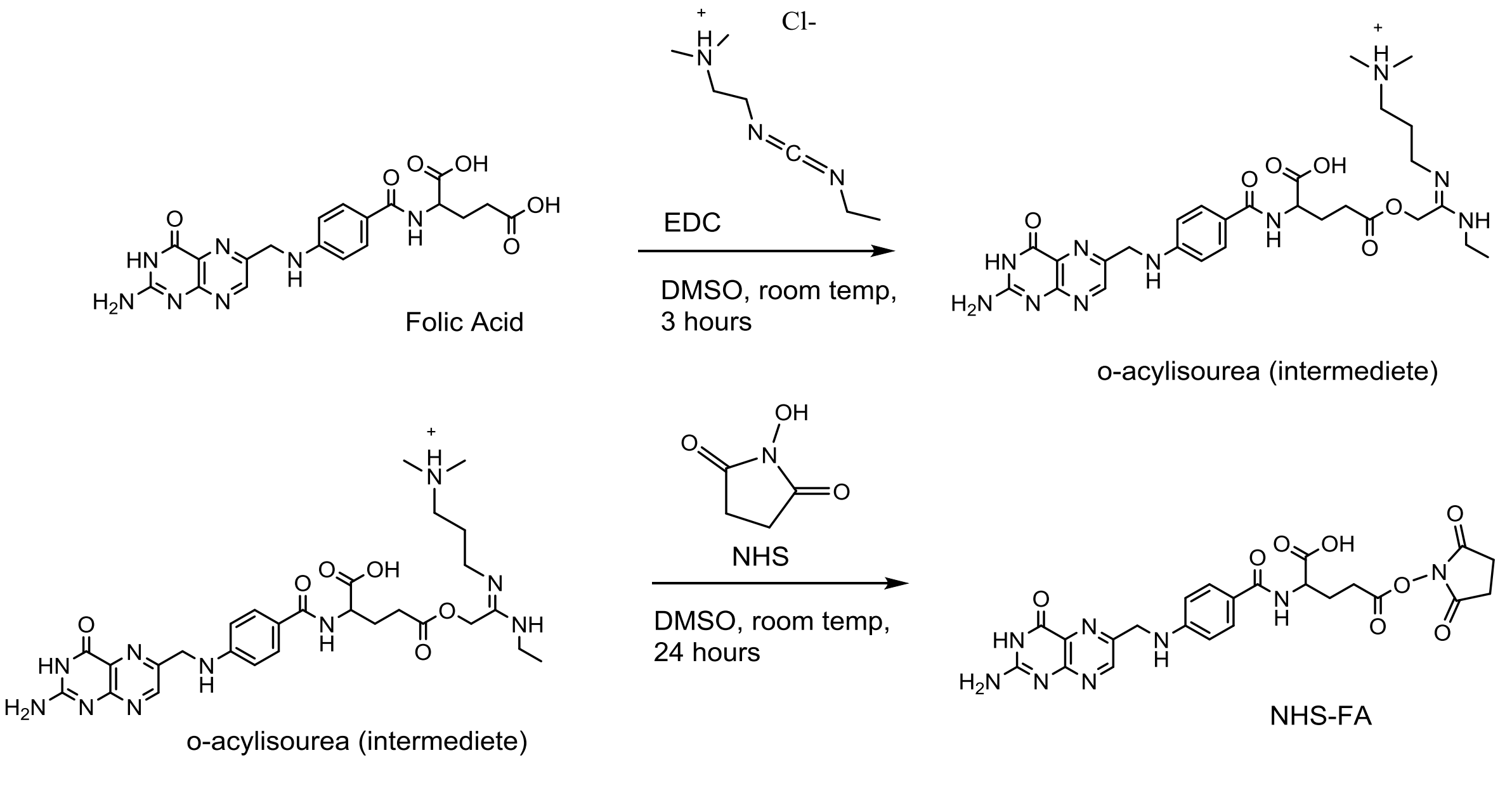

Figure S1. Scheme of the proposed reaction mechanism for the formation of APTMS-FA

APTMS-PEG-N_3 was prepared by adding 0.01 mmol APTMS into 0.5 mL of dry DMSO containing 0.003 mmol-NHS-PEG_{5000}-N_3 and stirring for 3 hours. Afterwards, the solution was treated with 0.01 mmol NHS, and reaction was continued under the dark at room temperature for 18-24 hours. The reaction product was characterized by FTIR spectroscopy.

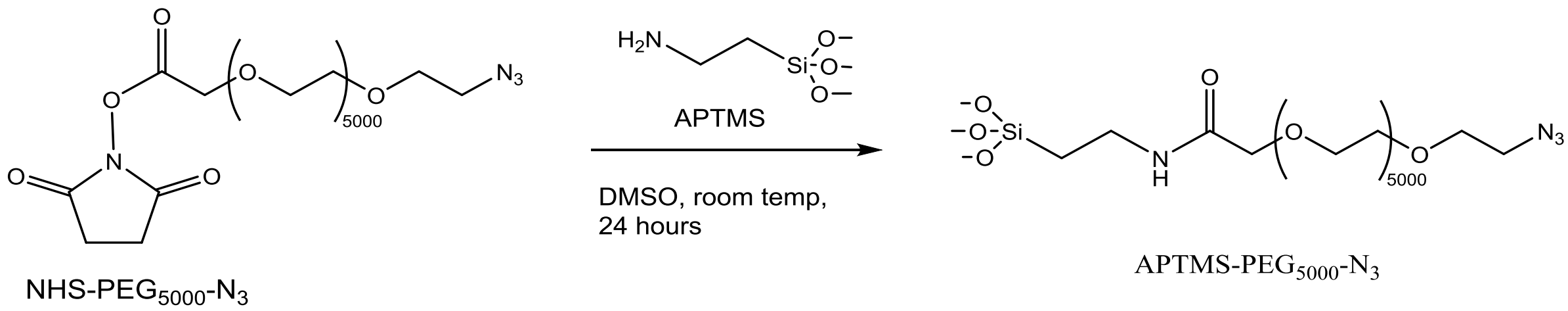

Figure S2. Scheme of the proposed reaction mechanism for the formation of APTMS-PEG-N_3

The next step in GdNPs functionalization was the dispersion of 10 mg of GdNPs in 5 mL of ethanol and 5 mL of Milli-Q water by sonication for about an hour to ensure proper dispersion. To this, APTMS and APTMS-PEG-N_3 conjugates were added, and the mixture was stirred at room temperature for 24 hours. The reaction suspension was centrifuged, washed several times with ethanol to remove unbound material, and then dried in a vacuum desiccator, yielding Gd-PEG-FA- N_3.

Characterization of Gadolinium Nanoparticle Before and After Functionalization :

The gadolinium nanoparticles, both before and after functionalization, were characterized using transmission electron microscopy (TEM), dynamic light scattering (DLS), Zeta Potential, UV-Vis spectroscopy, and Fourier-transform infrared (FTIR) spectroscopy.

Stability test : Stability testing was conducted by storing Gd-PEG-FA-N_3 nanoparticles under varying temperatures (25 °C and 4^oC) at pH 7.4 for 4 weeks. After storage, the nanoparticles were re-characterized using DLS to monitor changes in size and a UV spectrophotometer to analyze folic acid degradation.

Synthesis of Ho-DOTA-BCN Complex

0.01 mmol DOTA-BCN in 5 mL of Milli-Q water was mixed with 0.11 mmol $HoCl_3$ in 5 mL of Milli-Q water. The pH of the mixture was adjusted to 5.5 with 0.05 N NaOH, and the mixture was then refluxed at 60 °C for 8 hours. The reaction mixture was then stirred at room temperature overnight. To remove any unbound Ho(III), the obtained Ho-DOTA-BCN solution was passed through a Dowex 50W-X8 column and stirred for 4 hours. The presence of free Ho(III) ions in the solution was tested using xylenol orange; a purple color indicated a positive result. The concentration of Ho(III) in the final complex was determined using ICP-OES.

Monitoring of Click Reaction

Gd-PEG-FA-N_3 nanoparticles was dispersed in Milli-Q water and transferred to a cuvette, after which the Ho-DOTA-BCN complex was added. The reaction was monitored over a time course of 0-240 minutes using UV-Vis spectroscopy in the wavelength range 190–400 nm. Additionally, the click reaction between the Gd-FA-PEG-N_3 conjugate and the Ho-DOTA-BCN complex was also monitored under the same conditions.

Cell Culture

HeLa (human cervical adenocarcinoma) cells were cultured in Petri dishes in Roswell Park Memorial Institute (RPMI) medium containing 10% Fetal Bovine Serum (FBS) and 1% Penicillin at 37 °C with 5% CO_2 until 90% confluence was reached.

Cell Viability Click Reaction

The cytotoxicity of Gd-PEG-FA-N_3 nanoparticles with and without the biorthogonal click reaction with the Ho-DOTA-BCN complex was evaluated using the resazurin assay. HeLa cells (1.7×10^4 cell per well) in 100 µL of RPMI medium were seeded into 96-well plates and incubated at 37 °C with 5% CO_2 until 80% confluency was reached (approximately 24h). Subsequently, the medium in each well was replaced with medium containing functionalized Gd-PEG-FA-N_3 nanoparticles at varying concentrations (8-132 µg/mL), and the wells were incubated at 37 °C with 5% CO_2 for 24 h. After initial incubation of Gd-PEG-FA-N_3 nanoparticles, Ho-DOTA-BCN complexes were added at variation concentrations (0, 10, 50, 100, 150, 200, 250, 300 µg/mL) and incubated for an additional 24 hours to assess the effect of the in situ click reaction. Subsequently, 100 µL of resazurin solution was added to each well, and the cells were incubated for an additional 2 hours at 37 °C with 5% CO_2. Absorbance was measured at 570 nm using a microplate reader (n=4).

Statistical Analysis

Statistical analysis was conducted using one-way ANOVA followed by Dunnet Test (GraphPad Prism 10.5.0). Statistical significance was defined as $p < 0.05$ and expressed as mean ± standard deviation (SD) from 4 independent experiment (four replicates each).

Table S1. Stability Test Size of Gd-FA-PEG-N_3 nanoparticles Using DLS (n=3)

Weeks	Particles Size (nm)	
	25°C	4°C
0	7,9 ± 0,72	
1	8,73 ± 1,27	10,20 ± 1,32
2	8,47 ± 1,87	9,80 ± 1,13
3	11,23 ± 1,35	9,93 ± 0,76
4	71,83 ± 6,54	57,40 ± 1,39

Table S2. Result of folic acid stability test using UV spectrophotometer

Weeks	Folic Acid Concentration (x10^3 mmol)	
	**25°C**	**4°C**
0		
1	1.16 ± 0.18	1.17 ± 0.19
2	1.00 ± 0.17	0.98 ± 0.10
3	0.98 ± 0.10	0.96 ± 0.13
4	0.66 ± 0.16	0.77

Table S3. T-test of particle size stability

At 4°C storage

No	Comparison	T_test	p_value	Conclusion
1	Week0 vs Week1	-2.096693	0.170958069	Not Significant
2	Week0 vs Week2	-3.590662	0.069565997	Not Significant
3	Week0 vs Week3	-5.545455	0.031013339	Significant
4	Week0 vs Week4	-77.942286	0.000164568	Significant

At room temperature (25°C)

No	Comparison	T_test	p_value	Conclusion
1	Week0 vs Week1	-1.2453387	0.339123679	Not Significant
2	Week0 vs Week2	-0.3719458	0.74564461	Not Significant
3	Week0 vs Week3	-3.8461538	0.06143618	Not Significant
4	Week0vs Week4	-16.1023037	0.003834602	Significant

Table S4. T-test of folic acid degradation

At 4^oC storage

No	Comparison	T_test	p_value	Conclusion
1	Week0 vs Week1	51.961524	0.000370165	Significant
2	Week0 vs Week2	8.487049	0.013600524	Significant
3	Week0 vs Week3	12.619227	0.006221098	Significant
4	Week0 vs Week4	2.005533	0.182752618	Not Significant

At room temperature (25^oC)

No	Comparison	T_test	p_value	Conclusion
1	Week0 vs Week1_	26.846788	0.001384563	Significant
2	Week0 vs Week2_	27.135463	0.00135532	Significant
3	Week0 vs Week3_	8.487049	0.013600524	Significant
4	Week0 vs Week4	35.074029	0.000811894	Significant
